# Distribution and ecological preferences of *Ovalona karelica* (Stenroos, 1897) (Crustacea, Branchiopoda, Cladocera) in Volga River Basin (European Russia)

**DOI:** 10.3897/BDJ.13.e155864

**Published:** 2025-09-16

**Authors:** Dmitriy Gavrilko, Artem Sinev, Vyacheslav Zhikharev

**Affiliations:** 1 Lobachevsky State University of Nizhny Novgorod, Nizhni Novgorod, Russia Lobachevsky State University of Nizhny Novgorod Nizhni Novgorod Russia; 2 Department of Invertebrate Zoology, Biological Faculty, Lomonosov Moscow State University, Moscow, Russia Department of Invertebrate Zoology, Biological Faculty, Lomonosov Moscow State University Moscow Russia; 3 Lobachevsky State University of Nizhny Novgorod, Nizhny Novgorod, Russia Lobachevsky State University of Nizhny Novgorod Nizhny Novgorod Russia

**Keywords:** Cladocera, rare species, ecology, macrophytes, lakes, rivers

## Abstract

The distribution and ecological preferences of the rare water flea *Ovalona karelica* (Stenroos, 1897) (Crustacea, Branchiopoda, Cladocera) were analysed for the first time for European Russia. The species was found in different types of waterbodies, such as floodplain lakes of the Kerzhensky State Nature Biosphere Reserve, Nizhny Novgorod City and mouths of rivers inflowing into reservoirs. *Ovalona karelica* inhabits waters with wide ranges of pH, salinity, oxygen content and trophicity. Development of semi-submerged macrophytes (*Stratiotes*, *Hydrocharis*, *Salvinia*) is the main factor influencing occurrence of the species and its abundance.

## Introduction

Water fleas (Cladocera) of Russia are intensively studied and data on species distribution and ecology are accumulating rapidly. Over the last 150 years, reviews on many species of Cladocera with a wide distribution in the Palaearctic have been published (Smirnov 1971, Smirnov 1976, Sinev 2009, Kotov 2016, Korovchinsky 2018, Korovchinsky et al. 2021b). However, many rare species with restricted ranges are studied insufficiently. This knowledge can be important, however, for general ecological as well as palaeolimnological studies (Ibragimova et al. 2025). Such species often are characterised by their association with specific habitats. For example, *Holopedium gibberum* Zaddach, 1855 is associated with lakes with low calcium content (Korovchinskiy 2004, Zhikharev et al. 2019), *Acantholeberis curvirostris* (O.F. Müller, 1776) with *Sphagnum* bogs (Fryer 1993) and *Drepanothrix dentata* (Eurén, 1861) is associated with the zone between semi-submerged plants and plants with floating leaves (Smirnov 1976). One example of such rare species is *Ovalona karelica* (Stenroos, 1897) (Anomopoda, Chydoridae, Aloninae).

The morphology of *O. karelica* was recently studied by Van Damme et al. (2011) and Sinev and Gavrilko (2020). It is a rather distinctive species, clearly different from other European Aloninae of similar habitus in that it has a very small basal spine on the post-abdomen. It was described as a member of genus *Alona* Baird, 1843 and recently transferred in *Ovalona* (Sinev 2015).

According to Błedzki and Rybak (Bledzki and Rybak 2016), *O. karelica* is a relict glacial species with a limited distribution in the Palaearctic. It is one of the rarest European Aloninae species and its ecology is poorly studied (Van Damme et al. 2011). Occurences of the species are reported for the countries of northern and eastern Europe and in Germany and Denmark (Bledzki and Rybak 2016). *Ovalona karelica* occurs in dense vegetation in slightly acidic waters (Flössner 2000), in bogs, oxbow lakes and shallow lakes (Hudec 2010) and in lakes on muddy bottoms with plant remains and *Hydrochariton* sp. roots (Van Damme et al. 2011). It has been recorded in *Sphagnum* thickets in a dystrophic lake in Poland with pH 4.85-5.22 (Kuczynska-Kippen 2008).

In Russia, *O. karelica* was recorded in a few localities in Karelia, Yamal Peninsula and Taymyr Peninsula (Smirnov 1971, Sinev 2002). In the middle of the 20^th^ century, N.N. Smirnov found *O. karelica* in the coastal area of Rybinsk Reservoir and at the mouth of the Micha River in the Gorky Reservoir. The abundance of the species was low and did not exceed 8,000 specimens per m^3^ (Smirnov 1963). In 2017, *O. karelica* was found in thickets of *Stratiotes aloides* L., 1753 in rivers of Nizhny Novgorod City (Sinev and Gavrilko 2020), as well as in the mouth of Kerzhenets River (Gavrilko et al. 2020). In 2021, the species was found in floodplain lakes of the FGBU Khopersky State Reserve in thickets of *Typha angustifolia* L., 1753, *Potamogeton lucens* L., 1753 and *Ceratophyllum* sp. at extremely low abundance (< 1000 sp./m^3^) (Fedyaeva and Fedyaev 2022). These reports did not provide detailed information on the environmental conditions of the species habitat.

The aim of this study was to investigate distribution and ecological preferences of *O. karelica* in waterbodies of European Russia.

## Material and methods

**Figure 1.**
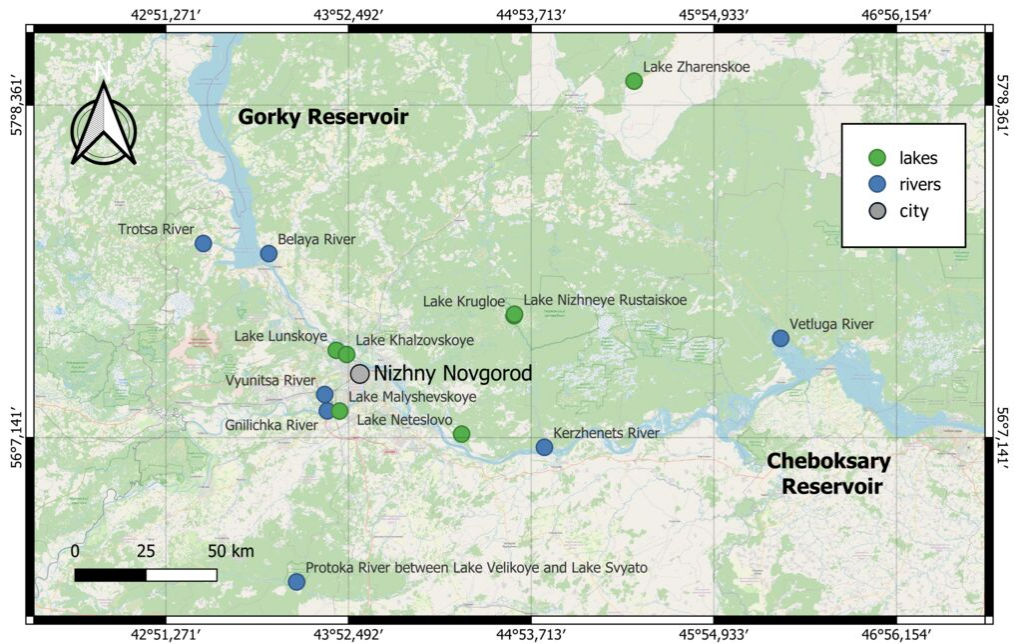
A map with the location of the explored waterbodies.

Our studies were conducted in 2017-2024 on 14 waterbodies (seven lakes and seven watercourses) located in the Volga River Basin. The rivers Viunitsa, Gnilichka and floodplain lakes Lunskoye, Khalzovskoye and Malyshevskoye are located within the city limits of Nizhny Novgorod (Nizhny Novgorod Region, Central Russia) (Fig. 1). The Belaya and Trotsa Rivers are the tributaries of the Gorky Reservoir, the Kerzhenets and Vetluga Rivers are tributaries of the Cheboksary Reservoir. The floodplain lakes Krugloe and Nizhneye Rustaiskoe are located in the territory of the Kerzhensky State Nature Biosphere Reserve (Shurganova et al. 2022). The watercourse connecting lakes Velikoye and Svyato is part of the Pustynskoye lake-river system. Lakes Zharenskoye and Neteslovo are located on the left bank of the Cheboksarskoye Reservoir in the Nizhny Novgorod Volga Region. Geographical coordinates of studied localities are given in Table 1.

The territory of the left bank of the Volga is characterised by a rather cold and humid climate. Precipitation falls annually 550-600 mm and the average annual air temperature is 2.3^o^C - 2.9^o^C. The climate of the right bank of the Volga is drier and warmer than on the left bank. Precipitation per year is 450-550 mm and the average annual air temperature is 3.4^o^C - 3.8^o^C (Agafonov 1974). These conditions are permanent.

These habitats were sampled as part of an extensive sampling programme of the Volga River Basin during 2017-2024. In total, over 1150 waterbodies were sampled in the Moscow Area, Nizhny Novgorod Area, Ivanovo Area, Kostroma Area, Mari El Republic, Republic of Tatarstan and Chuvash Republic. *Ovalona karelica* was found in only 33 habitats.

Crustacean samples were collected in July – early August in macrophyte thickets by bucketing 25-50 l of water through a plankton net (nylon sieve with a mesh diameter of 70 µm). Samples were preserved with 40% formalin, adjusted to 4% concentration in the sample. Samples were studied using Carl Zeiss Stemi 2000C binocular microscope. Detailed morphological analysis was performed under an Olympus CX43 microscope. Abiotic environmental conditions were measured during the sampling. Water transparency was determined by Secchi white disc. Water temperature, pH and conductivity were measured with a YSI Pro 1030 multiparameter probe. Dissolved oxygen content and chlorophyll a concentration were measured with an Aquared AP2000 multiparameter probe. When specifying the taxonomic affiliation of zooplankton, we used proper manuals and guides (Alekseev and Tsalolikhin 2010, Korovchinsky et al. 2021b).

One-way ANOVA (followed by t-test post-hoc comparisons with Holm correction) was applied to test for differences in density of *O. karelica* in different types of macrophytes and types of waterbodies. Single-predictor linear regression models (separately for each environmental factor) were applied to test the effects of the environmental factors on the density of *O. karelica*. The assumptions of the regression analysis, normality and homogeneity of variance were initially verified using the Shapiro–Wilk and Levene tests, respectively. Linear regression models were verified using ANOVA (Borcard et al. 2011, Legendre and Legendre 2012). Statistical analyses were performed using R open-source software ("dplyr" package).

## Results

The results of measuring abiotic environmental parameters are presented in Table 1.

**Figure 2.**
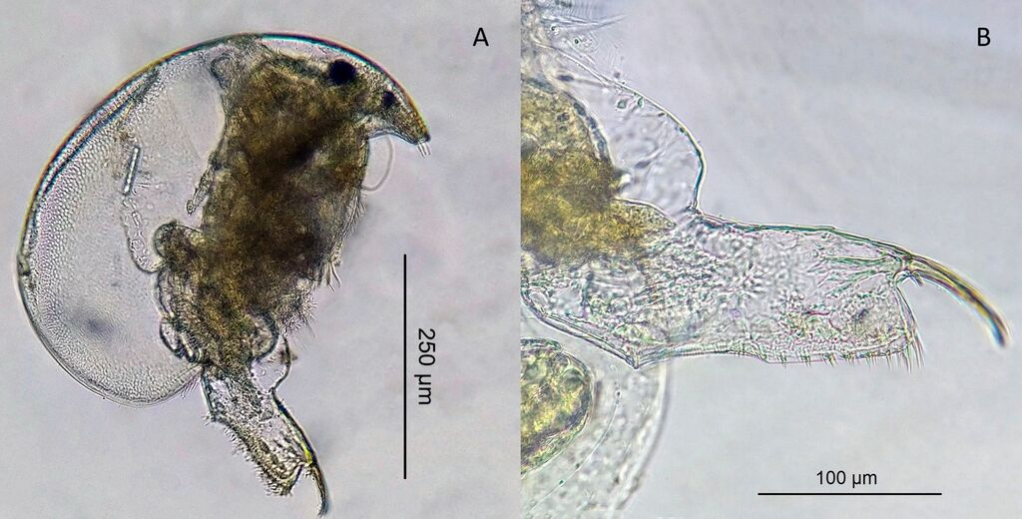
Lateral view (A) and post-abdomen (B) of a parthenogenetic female of *Ovalona karelica* (Stenroos, 1897) from Lake Khalzovskoe.

**Figure 3.**
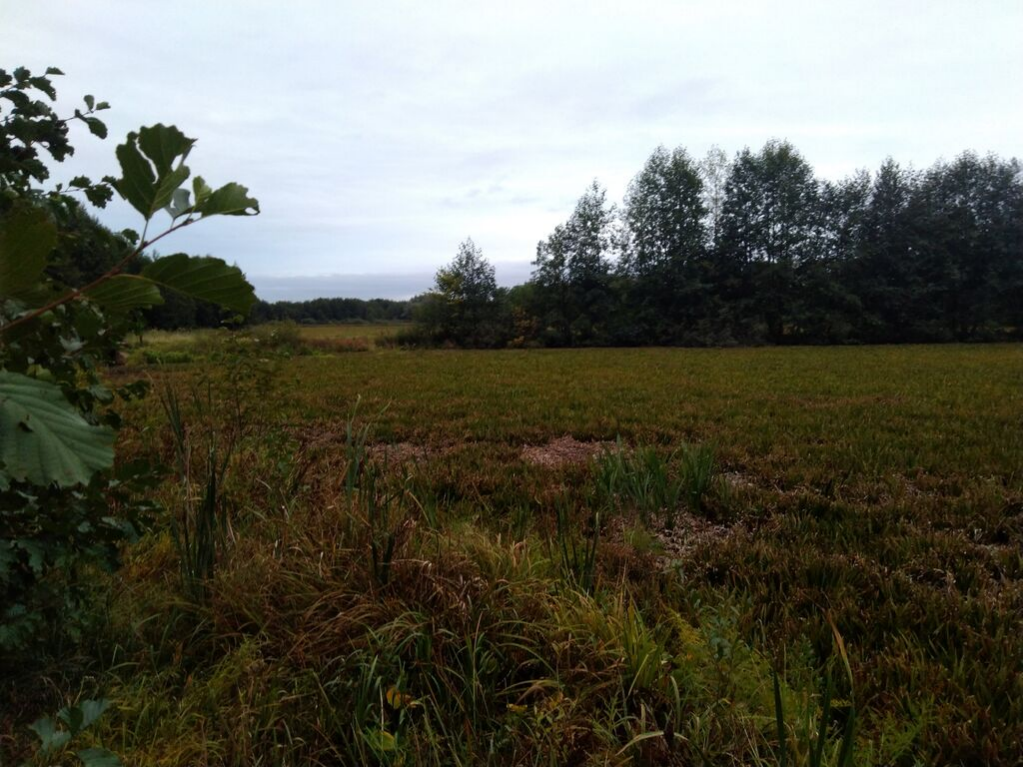
*Stratiotes aloides* thickets from Gnilichka River, Nizhny Novgorod Region, Russia. 7.08.2019. Gavrilko D.E.

**Figure 4.**
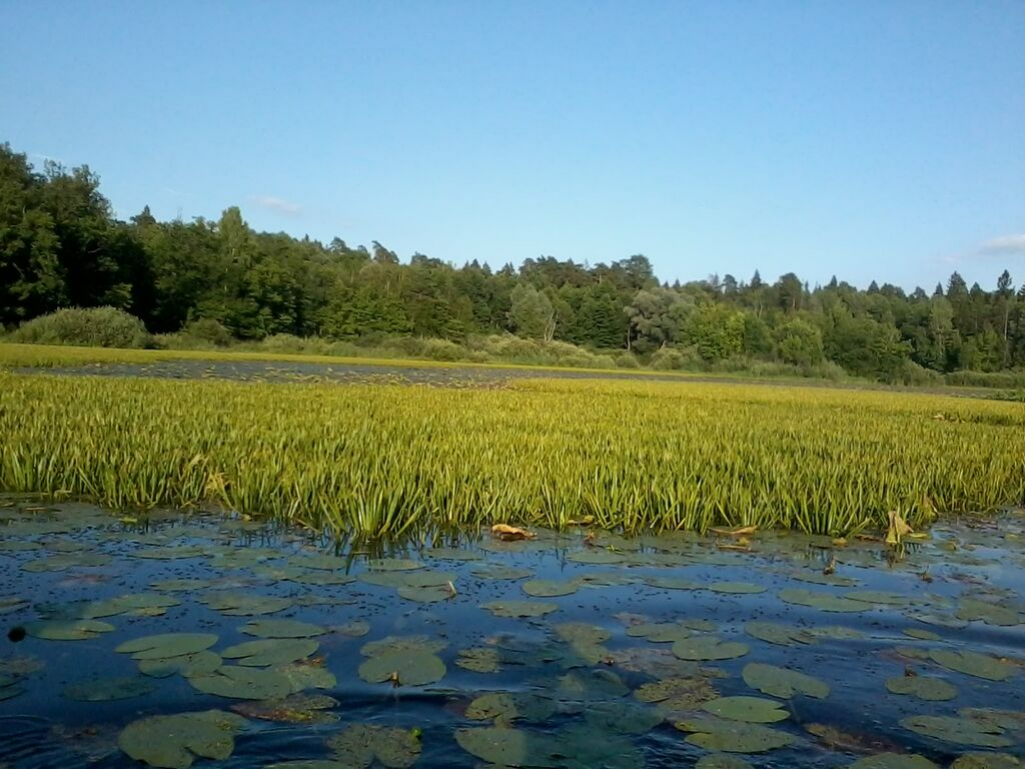
*Stratiotes aloides* thickets from Protoka River between Lake Velikoye and Lake Svyato, Nizhny Novgorod Region, Russia. 16.07.2018. Gavrilko D.E.

**Figure 5.**
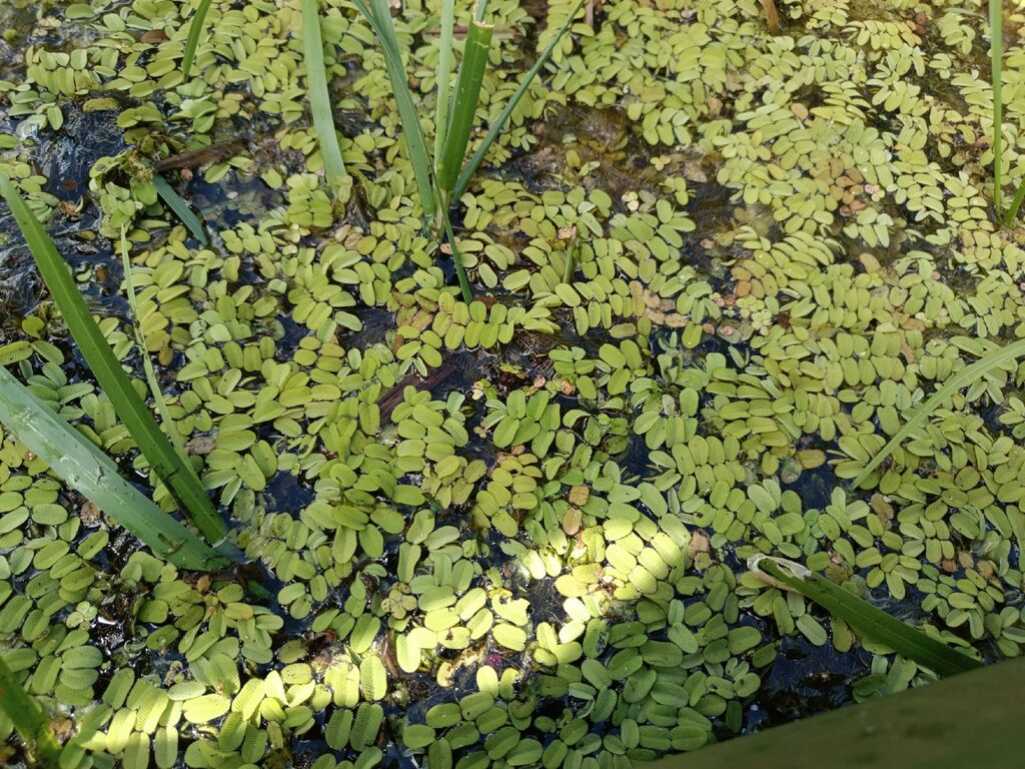
*Salvinia natans* thickets from Lake Khalzovskoye, Nizhny Novgorod Region, Russia. 25.07.2024. Gavrilko D.E.

**Figure 6.**
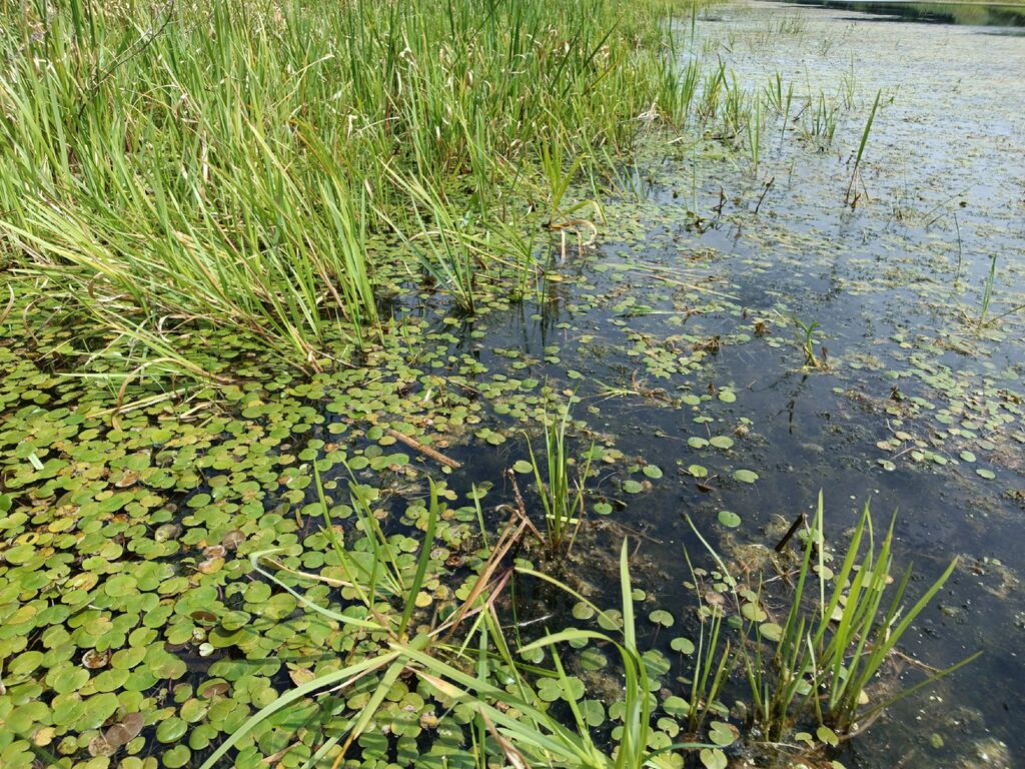
*Hydrocharis morsus-ranae* thickets from Lake Malyshevskoye, Nizhny Novgorod Region, Russia. 26.07.2024. Gavrilko D.E.

The appearance of *Ovalona karelica* and some habitats are presented in the figures (Figs 2, 3, 4, 5, 6).

Almost all surveyed biotopes are located in Nizhny Novgorod Oblast, except for the mouth of the Vetluga River, which is located in the Republic of Mari El. Compared to previous studies (Gavrilko et al. 2020), *O. karelica* was found in seven lakes and three rivers for the first time. The species inhabits water bodies with pH from slightly acidic to slightly alkaline (6.10-8.54) (Table 1) and a wide range of water conductivity (19-402 μS/cm). *Ovalona karelica* habitats varied greatly in dissolved oxygen content of water, varying from 0.1 to 10.4 mg/l. The trophic status of river and lake waters varied from mesotrophic to eutrophic.

In most habitats, the quantitative development of *O. karelica* was low and did not exceed 0.5 thousands ind./m^3^ (Table 2). *O. karelica* was low and did not exceed 0.5 thousands ind./m^3^ (Table 2). The exceptions were thickets of *Stratiotes* in the pond extension of the Gnilichka River, where the density reached 17.6 thousands ind./m^3^ and thickets of *Stratiotes* in the mouth area of the Kerzhenets River, where the densities were 4.0 and 12.0 thousands ind./m^3^.

In the majority of studied habitats, *O. karelica* did not dominate amongst Cladocera. The exception was the site of pond expansion of the Gnilichka River, where the share of the species was 15.6 % of the total cladoceran abundance (Table 2). This habitat is characterised by dense thickets of *Stratiotes* and low concentration of dissolved oxygen in the water (Table 1).

In the studied rivers, *O. karelica* was most often found in *Stratiotes* thickets (Table 1). In the urban Rivers Vyunitsa and Gnilichka, its mass development is confined to pond extensions. In the Kerzhenets and Vetluga Rivers, extensive stands of *Stratiotes* are concentrated in estuaries. In such habitats, slow current velocity, low depth and significant water warming in summer lead to extensive overgrowing of the water area with *Stratiotes*. In lakes, occurrences of *O. karelica* are confined to thickets of helophytes (*Carex*, *Glyceria*) or mixed thickets of helophytes with *Hydrocharis*, *Salvinia* and *Stratiotes*. The species was not found in pure thickets of *Nuphar lutea* (L.) Sm. or *Elodea canadensis* Michx. in the studied lakes and rivers. Presence of *O. karelica* in a particular waterbody or watercourse clearly is correlated with the development of the abovementioned macrophyte species.

**Figure 7.**
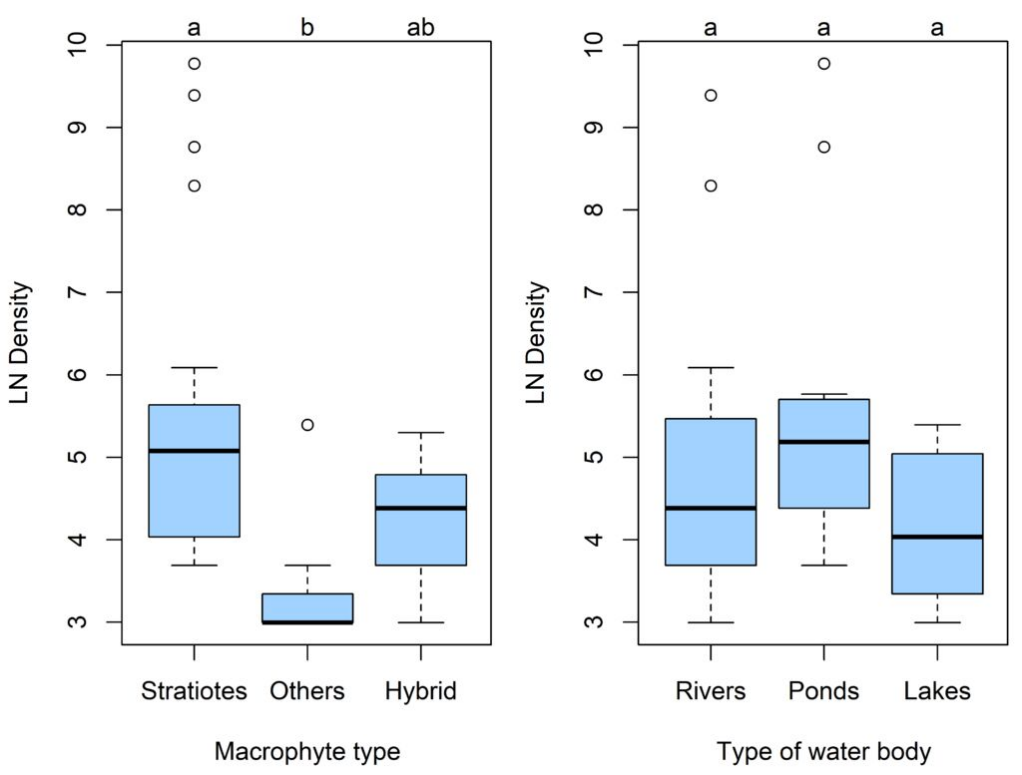
Boxplots of the density *O. karelica* in different macrophyte type and waterbodies. Compact letter displays were added to indicate significant differences.

The ANOVA (F_2.36_ = 4.52, p = 0.017) conducted between different types of macrophytes showed that the density of *O. karelica* is significantly higher in the thickets of *Stratiotes* than in other thickets. No differences were found in the density of *O. karelica* between *Stratiotes* and hybrid thickets with *Hydrocharis* and *Salvinia*. We found no statistically (F_2.36_ = 1.67, p = 0.20) significant differences in *O. karelica* density between different types of waterbodies. However, the median density in ponds is higher than in lakes and rivers (Fig. 7).

Regression analysis showed a significant relationship between the density of O. karelica and water transparency. The density of O. karelica decreased with increasing transparency. This suggests that the crustacean prefers more turbid waters. Low transparency is facilitated by dense thickets of *Stratiotes* due to the accumulation of detritus under the plants (Table 3).

## Discussion

Our study shows that *O. karelica* in the Volga River Basin has a wider area of distribution than was presumed previously (Korovchinsky et al. 2021b). The species inhabits macrophyte thickets in floodplain lakes, ponds on dammed rivers and estuaries of rivers inflowing into reservoirs. Species habitats have rather strong variability of abiotic conditions.

*Ovalona karelica* was found in a mesotrophic lake with low water mineralisation and in several eutrophic floodplain lakes. The species inhabits lakes of Kerzhensky Reserve with slightly acidic waters (pH 6.10-6.64) and low mineralisation (57-117 μS/cm), as well as in the estuaries of lakes inflowing into reservoirs and lakes with alkaline waters (pH 8.15-8.54) and significant mineralisation (365-402 μS/cm). The species reached the highest density in the eutrophic pond on an urban river. Finding the crustacean in dystrophic lakes with low pH and mineralisation (Kuczynska-Kippen 2008) and eutrophic rivers and lakes indicates a wide plasticity of the species in relation to these factors. *Ovalona karelica* occurred at wide range of oxygen concentrations, including rather low (Table 1). The highest density and the greatest presence of the species within the community (Table 2) were attained at the habitat with the lowest oxygen concentrations (0.1-1.46 mg/l). Dissolved oxygen content in water is one of the limiting factors for aquatic animals. Some phytophilous Cladocera are oxyphilous: for example, *Sida crystallina* (O.F. Müller, 1776) and *Eurycercus lamellatus* (O.F. Müller, 1776) and cannot tolerate low oxygen concentrations (Korovchinsky et al. 2021a). The tolerance for low oxygen concentrations in *O. karelica* appears to be a competitive advantage over other Cladocera species. When oxygen concentration decreases, *O. karelica* increases its density taking advantage of the reduced density of other cladocerans and gains access to unoccupied resources.

The presence of *O. karelica* in the studied waterbodies correlates with the presence of specific macrophytes. In Eastern Europe, the species is associated with *Sphagnum* mosses (Flössner 2000, Kuczynska-Kippen 2008) and *Hydrochariton* thickets (Hudec 1986). In the studied habitats, the crustacean was more commonly found in *Stratiotes* thickets, where it attained highest densities. In mixed thickets, its occurrence was recorded in the presence of *Stratiotes*, *Salvinia* or *Hydrocharis* only. These three plant species have similar morphology, being semi-submerged macrophytes with leaves floating on the surface and roots submerged in water. The presence of such thickets allows *O. karelica* to live amongst submerged roots. Occurrence in such specific habitats explains the rarity of this species. Such marginal habitats are rarely sampled during zooplankton studies. Rare species with specific adaptations may be abundant and dominant in only certain, rather narrow ecological niches and habitats (Hessen and Walseng 2008). *Ovalona karelica* appear to be an example of species with exactly this evolutionary strategy, being a specialist adapted to a very specific type of habitat.

The performed statistical analysis confirms our assumptions. The increase in the density of *O. karelica* in *Stratiotes* thickets indicates favourable habitat conditions for it. Dense *Stratiotes* thickets lead to accumulation of detritus on them. This causes an increase in water turbidity and a decrease in transparency. Regression analysis confirmed a decrease in the density of *O. karelica* with increasing transparency. Such conditions do not form in single-species thickets (e.g. *Carex*, *Gliceria*, *Equisetum*). In helophytes, the main part of the plant is above water and the roots are in the bottom. As a result, a weak substrate attachment for the crustacean is formed in such thickets. The density of *O. karelica* is very low in such thickets (Others on boxplots).

The number of cladocerans varied in the studied biotopes from 14 to 35, reaching a maximum in the *Stratiotes* in the Kerzhenets and Vetluga Rivers. In most of the *Stratiotes* thickets of rivers and lakes, Ceriodaphnia pulchela Sars, 1862 dominated. In the *Stratiotes* thickets of ponds, *Coronatella rectangula* Sars, 1862 and *Alonella exigua* (Lilljeborg, 1901) dominated. In the helophyte thickets, *C. rectangulia* and *A. exigua* and *Chydorus sphaericus* (O.F. Muller, 1785) dominated. Thus, the most ecologically attractive species in *O. karelica* are representatives of the Chydoridae, which lead a creeping lifestyle.

Our data suggest that studies of specific habitats, such as *Sphagnum* thickets, thickets of semi-submerged and submerged macrophytes, mixed thickets of macrophytes of different ecological groups, are crucial for revealing full biodiversity of substrate-associated Cladocera. Better understanding of ecology and limited ranges of distribution of rare species can be achieved by analysis of abiotic conditions of their habitats. This can be crucial information for ecological as well as palaeolimnological studies that include cladocerans.

## Conclusions

The distribution and ecological preferences of *O. karelica* were analysed for the first time for the territory of European Russia. The species was found in different types of waterbodies, such as floodplain lakes of the Kerzhensky Reserve, floodplain lakes of Nizhny Novgorod City and estuaries of tributary rivers of reservoirs. The species is present in a wide range of acidity, mineralisation and trophic conditions. The highest density of the crustacean was recorded in *Stratiotes* thickets of a eutrophied pond extension of a river. The tolerance of *O. karelica* to low oxygen content in water was noted. We believe that *O. karelica* is an inhabitant of the rhizosphere of semi-submerged macrophytes. The limited distribution of this species in European Russia may be related to habitat specificity.

## Figures and Tables

**Table 1.** Water conditions and dominant macrophyte species in the studied biotopes.

Water body	Coordinates (N, E)	WT, ^o^C	pH	EC, μS/cm	DO, mg/l	Chl-a, mkg/l	SD, m	Dominant macrophyte
Kerzhenets River 2017	56.091674, 44.962814	22.7	6.96	111	6.49	NA	0.5	*Stratiotes aloides*+*Nuphar lutea*
Kerzhenets River 2022	56.085643, 44.963219	27.3	8.13	194	7.99	6.6	0.9	*N. lutea*+*Potamogeton natans*+*Sparganium* sp.
	56.089195, 44.968739	28.3	8.15	171	8.19	12.9	0.4	*S. aloides*
	56.091897, 44.960453	25.7	8.22	168	6.74	8.5	0.3	*S. aloides*
	56.091453, 44.941249	27.1	8.34	191	7.81	5.1	0.4	*S. aloides*
Vyunitsa River 2017	56.250078, 43.742391	22.0	7.13	341	6.75	NA	0.4	*S. aloides*
	56.245891, 43.743170	25.4	7.46	365	5.82	NA	0.3	*S. aloides*
	56.220471, 43.720587	20.7	7.32	319	NA	NA	0.5	*S. aloides*
Gnilichka River 2017	56.213382, 43.746679	23.6	7.40	286	NA	NA	0.5	*S. aloides*
	56.209321, 43.753670	23.0	7.00	324	NA	NA	0.4	*S. aloides*
	56.205236, 43.756818	22.4	6.90	325	0.10	NA	0.5	*S. aloides*
	56.205270, 43.756927	22.4	6.92	307	1.46	NA	0.3	*S. aloides*
	56.202471, 43.756306	23.6	7.21	321	1.70	NA	0.3	*S. aloides*
	56.195305, 43.743675	24.3	7.45	316	2.62	NA	0.5	*S. aloides*
Protoka River between Lake Velikoye and Lake Svyato 2018	55.667258, 43.586353	22.4	7.65	160	NA	NA	0.5	*S. aloides*
	55.667291, 43.586283	23.1	7.53	144	NA	NA	0.4	*S. aloides*
	55.667312, 43.586246	22.5	7.50	144	NA	NA	0.4	*S. aloides*
	55.667321, 43.586221	22.7	7.52	145	NA	NA	0.4	*S. aloides*
	55.667341, 43.586189	22.8	7.53	146	NA	NA	0.6	*S. aloides*
	55.667358, 43.586146	22.9	7.52	145	NA	NA	0.6	*S. aloides*
Vetluga River 2020	56.415460, 46.237744	20.6	7.37	204	6.50	6.2	0.5	*S. aloides*
Vetluga River 2021	56.429478, 46.278870	27.0	8.30	345	9.01	21.3	0.4	*S. aloides*
Trotsa River 2021	56.717498, 43.059559	20.1	6.60	165	7.36	4.5	0.5	*N. lutea*+*Sparganium* sp.
	56.717492, 43.064243	21.6	6.80	165	7.20	7.16	0.5	*Equisetum fluviatile*
Belaya River 2022	56.687223, 43.429567	23.7	7.93	200	5.51	18.46	0.7	*Sagittaria sagittifolia*+ *Salvinia natans*+*Sparganium* sp.
Lake Zharenskoe 2019	57.212075, 45.470434	19.3	6.97	19	NA	NA	0.4	*Carex* sp.
Lake Krugloe 2021	56.495686, 44.797231	21.9	6.10	58	4.82	NA	0.4	*P. natans*
Lake Krugloe 2022	56.496244, 44.799083	26.8	6.64	57	5.91	NA	0.3	*Alisma plantago-aquatica*+*Sparganium* sp.
Lake Nizhneye Rustaiskoe 2022	56.500062, 44.802044	24.4	6.53	117	6.02	NA	0.3	*Glyceria maxima*
Lake Neteslovo 2023	56.132142, 44.505644	NA	NA	NA	NA	NA	NA	*S. aloides*+*Hydrocharis morsus-ranae*
Lake Lunskoye 2023	56.389250, 43.805529	22.2	8.54	402	10.4	18.8	0.4	*G. maxima*+*H. morsus-ranae*
Lake Khalzovskoye 2024	56.376702, 43.864003	23.8	7.00	187	6.40	17.7	0.4	*G. maxima+S. natans*
Lake Malyshevskoye 2024	56.201555, 43.824907	24.1	8.20	224	7.35	20.36	0.3	*G. maxima*+*H. morsus-ranae*

Note. WT - water temperature, EC - electrical conductivity, DO - dissolved oxygen concentration, Chl-a - chlorophyll a concentration, SD - water transparency.

**Table 2.** Density of *O. karelica* in the studied water-bodies.

Waterbody and year of sampling	Coordinates	Density, ×10^3^ ind./m^3^	% of the total Cladocera density
Kerzhenets River 2017	56.091674, 44.962814	0.12	0.91
Kerzhenets River 2022	56.085643, 44.963219	0.02	0.0001
	56.089195, 44.968739	4.0	0.016
	56.091897, 44.960453	12.0	0.032
	56.091453, 44.941249	0.14	0.001
Vyunitsa River 2017	56.250078, 43.742391	0.08	0.14
	56.245891, 43.743170	0.08-0.32	0.073-0.75
	56.220471, 43.720587	0.04-0.44	0.15-1.25
Gnilichka River 2017	56.213382, 43.746679	0.04-0.08	0.02-0.04
	56.209321, 43.753670	0.22	0.25
	56.205236, 43.756818	0.2	6.10
	56.205270, 43.756927	6.4-17.6	7.77-15.6
	56.202471, 43.756306	0.16	0.17
	56.195305, 43.743675	0.04	0.05
Protoka River between Lake Velikoye and Lake Svyato 2018	55.667258, 43.586353	0.04	0.024
	55.667291, 43.586283	0.08	0.072
	55.667312, 43.586246	0.04	0.035
	55.667321, 43.586221	0.04	0.021
	55.667341, 43.586189	0.16	0.25
	55.667358, 43.586146	0.28	0.58
Vetluga River 2020	56.415460, 46.237744	0.2	0.25
Vetluga River 2021	56.429478, 46.278870	0.04	0.01
Trotsa River 2021	56.717498, 43.059559	0.02	0.03
	56.717492, 43.064243	0.02	0.04
Belaya River 2022	56.687223, 43.429567	0.02	0.0001
Lake Zharenskoe 2019	57.212075, 45.470434	0.04	0.29
Lake Krugloe 2021	56.495686, 44.797231	0.02	0.001
Lake Krugloe 2022	56.496244, 44.799083	0.22	0.003
Lake Nizhneye Rustaiskoe 2022	56.500062, 44.802044	0.02	0.0001
Lake Neteslovo 2023	56.132142, 44.505644	0.2	0.001
Lake Lunskoye 2023	56.389250, 43.805529	0.04	0.003
Lake Khalzovskoye 2024	56.376702, 43.864003	0.08	0.01
Lake Malyshevskoye 2024	56.201555, 43.824907	0.12	0.13

**Table 3.** Results of regression analysis of density *O. karelica* and environmental factors.

Environmental factors	R-squared	F-statistic	p-value
Water transparency	0.135	6.765	**0.013**
Dissolved oxygen concentration	0.081	3.114	0.091
Electrical conductivity	0.011	1.423	0.241
Water temperature	0.007	1.252	0.271
рН	-0.018	0.333	0.568
Chlorophyll a concentration	-0.087	0.117	0.739

## References

[B13331457] Agafonov S. M. (1974). Nature of the Gorky region.

[B12672758] Alekseev V. R., Tsalolikhin S. A. (2010). Taxonomic key of zooplankton and freshwaterzoobenthos of European Russia. Vol. 1. Zooplankton..

[B12672681] Bledzki L. A., Rybak J. I. (2016). Freshwater Crustacean Zooplankton of Europe: Cladocera & Copepoda (Calanoida, Cyclopoida). Key to species identification, with notes on ecology, distribution, methods and introduction to data analysis.

[B12911250] Borcard D, Gillet F, Legendre P (2011). Numerical Ecology with R.

[B12672689] Fedyaeva L. A., Fedyaev R. A. (2022). On the finding of the rare species *Ovalona
karelica* (Stenroos, 1897) (Branchiopoda: Anomopoda: Chydoridae) in the flood-plain lakes of the Khoper Nature Reserve. Transactions of Papanin Institute for Biology of Inland Waters RAS..

[B12672698] Flössner D. (2000). Die Haplopoda und Cladocera (ohne Bosminidae) Mitteleuropas.

[B12672706] Fryer G. O. (1993). The freshwater Crustacea of Yorkshire: A faunistic and ecological survey.

[B12672714] Gavrilko D. E., Zhikharev V. S., Ruchkin D. S., Zolotoreva T. V., Shurganova G. V. (2020). Cladocerans in the higher aquatic plantsplant’s thickets in European Russia, the inflows of Gorkovskay and Cheboksarsky reservoirs taken as examples. Zoologicheskii Zhurnal.

[B12672724] Hessen D. O., Walseng B. (2008). The rarity concept and the commonness of rarity in freshwater zooplankton. Freshwater Biology.

[B12672741] Hudec I. (1986). Further notes on *Alona
karelica* (Cladocera, Chydoridae) from East Slovakia. Věstník Československé společnosti Zoologické.

[B12672750] Hudec I. (2010). Anomopoda, Ctenopoda, Haplopoda, Onychopoda (Crustacea: Branchiopoda).

[B13331483] Ibragimova A. G., Seleznev D. G., Frolova L. A., Subetto D. A., Potakhin M. S., Belkina N. A., Kotov A. A. (2025). Cladoceran remains as a tool for reconstruction of past environmental conditions during the Late Pleistocene–Holocene in Central Karelia (NW Russia). Part I. Traditional quantitative analysis. Arthropoda Selecta.

[B12672766] Korovchinskiy N. M. (2004). Crustacean crustaceans of the Ctenopoda order of the world fauna (morphology, systematics, ecology, zoogeography).

[B12672774] Korovchinsky N. M. (2018). Cladocera: Ctenopoda. Families Sididae, Hopopedidae & Pseudopenilidae (Brachiopoda: Cladocera). Identification Guides to the plankton and benthos of Inland waters.

[B12672782] Korovchinsky N. M., Kotov A. A., Boikova O. S., Smirnov N. N. (2021). Cladocera (Crustacea: Cladocera) of Northern Eurasia..

[B12672790] Korovchinsky N. M., Kotov A. A., Sinev A. Y., Neretina A. N., Garibian P. G. (2021). Cladocera (Crustacea: Cladocera) of Northern Eurasia..

[B12672799] Kotov A. A. (2016). Faunistic complexes of the Cladocera (Crustacea, Branchiopoda) of Eastern Siberia and the Far East of Russia. Biology Bulletin.

[B12672808] Kuczynska-Kippen N. (2008). Spatial distribution of zooplankton communities between the *Sphagnum* mat and open water in a dystrophic lake. Polish Journal of Ecology.

[B12911242] Legendre P, Legendre L (2012). Numerical ecology.

[B13327815] Shurganova G. V., Zhikharev V. S., Gavrilko D. E., Zolotareva T. V., Kudrin I. A., Ilyin M. Yu., Bayanov N. G. (2022). Zooplankton of the Kerzhensky Reserve (annotated list of species). Flora and fauna of Reserves..

[B12672817] Sinev A. Y. (2002). A key to identifying cladocerans of the genus *Alona* (Anomopoda, Chydoridae) from the Russian European part and Siberia. Zoologicheskii Zhurnal.

[B12672826] Sinev A. Y. (2009). Discrimination between two sibling species of *Acroperus* (Baird, 1843) from the Palearctic (Cladocera: Anomopoda: Chydoridae). Zootaxa.

[B13331470] Sinev A. Yu. (2015). Revision of the pulchella-group of *Alona* s. lato leads to its translocation to *Ovalona* Van Damme et Dumont, 2008 (Branchiopoda: Anomopoda: Chydoridae). Zootaxa.

[B12672835] Sinev A. Y., Gavrilko D. E. (2020). Examples of rare benthic Cladocera: two phytophilous species of Aloninae (Cladocera, Anomopoda, Chydoridae) from european Russia. Zoologicheskii Zhurnal.

[B12672853] Smirnov N. N. (1963). On inshore Cladocera of the Volga Water Reservoirs. Hydrobiologia.

[B12672862] Smirnov N. N. (1971). Chydoridae of the world fauna. Fauna USSR. Rakoobraznie.

[B12672888] Smirnov N. N. (1976). Macrothricidae and Moinidae of the World Fauna. Fauna USSR. Rakoobraznie.

[B12672870] Van Damme K., Elias-Gutierrez M., Dumont H. J. (2011). Three rare European “*Alona*” taxa (Branchiopoda: Cladocera: Chydoridae), with notes on distribution and taxonomy. Annales de Limnologie.

[B12672879] Zhikharev V. S., Shurganova G. V., Kudrin I. A. (2019). Records of *Holopedium
gibberum* Zaddach, 1855 (Crustacea: Cladocera) in the floodplain lakes of the Kerzhensky Nature Reserve (Nizhny Novgorod region, Russia). Invertebrate Zoology.

